# Is Diabetes a Risk Factor for a Severe Clinical Presentation of Dengue? - Review and Meta-analysis

**DOI:** 10.1371/journal.pntd.0003741

**Published:** 2015-04-24

**Authors:** Nan Shwe Nwe Htun, Peter Odermatt, Ikenna C. Eze, Noémie Boillat-Blanco, Valérie D’Acremont, Nicole Probst-Hensch

**Affiliations:** 1 Department of Epidemiology and Public Health, Swiss Tropical and Public Health Institute, Basel, Switzerland; 2 University of Basel, Basel, Switzerland; 3 Infectious Diseases Service, Lausanne University Hospital, Lausanne, Switzerland; 4 Department of Ambulatory Care and Community Medicine, University of Lausanne, Lausanne, Switzerland; Pediatric Dengue Vaccine Initiative, UNITED STATES

## Abstract

**Background:**

The mean age of acute dengue has undergone a shift towards older ages. This fact points towards the relevance of assessing the influence of age-related comorbidities, such as diabetes, on the clinical presentation of dengue episodes. Identification of factors associated with a severe presentation is of high relevance, because timely treatment is the most important intervention to avert complications and death. This review summarizes and evaluates the published evidence on the association between diabetes and the risk of a severe clinical presentation of dengue.

**Methodology/Findings:**

A systematic literature review was conducted using the MEDLINE database to access any relevant association between dengue and diabetes. Five case-control studies (4 hospital-based, 1 population-based) compared the prevalence of diabetes (self-reported or abstracted from medical records) of persons with dengue (acute or past; controls) and patients with severe clinical manifestations. All except one study were conducted before 2009 and all studies collected information towards WHO 1997 classification system. The reported odds ratios were formally summarized by random-effects meta-analyses. A diagnosis of diabetes was associated with an increased risk for a severe clinical presentation of dengue (OR 1.75; 95% CI: 1.08–2.84, p = 0.022).

**Conclusions/Significance:**

Large prospective studies that systematically and objectively obtain relevant signs and symptoms of dengue fever episodes as well as of hyperglycemia in the past, and at the time of dengue diagnosis, are needed to properly address the effect of diabetes on the clinical presentation of an acute dengue fever episode. The currently available epidemiological evidence is very limited and only suggestive. The increasing global prevalence of both dengue and diabetes justifies further studies. At this point, confirmation of dengue infection as early as possible in diabetes patients with fever if living in dengue endemic regions seems justified. The presence of this co-morbidity may warrant closer observation for glycemic control and adapted fluid management to diminish the risk for a severe clinical presentation of dengue.

## Author Summary

Both dengue and diabetes have reached epidemic dimensions and pose a joint threat to a large proportion of populations in low- and middle-income countries. Dengue is no longer a disease primarily affecting children. Therefore the influence of non-communicable diseases such as diabetes, which are increasingly prevalent in adults, on the clinical presentation of a dengue episode becomes a public health priority. We conducted a systematic literature review to assess the available evidence on the effect of diabetes mellitus (DM) on the clinical presentation of dengue. The meta-analysis of published evidence combined with supporting biological evidence point to an increased risk for potentially life threatening symptoms of dengue among patients with diabetes. The current evidence is limited by statistical power and other study limitations and does not allow conclusions about a causal effect of diabetes. Yet, based on the currently available evidence, diabetes patients with fever and living in a dengue endemic region should seek confirmation of dengue infection as early as possible. Diabetes should be considered in the triage of patients for close observation and early intervention, which are challenges, particularly during dengue outbreaks. Timeliness of intervention is the most important factor averting serious complications and death in patients with acute dengue.

## Introduction

With low- and middle-income countries (LMIC) experiencing a growing chronic non-communicable disease (NCD) burden and a continuously high communicable disease (CD) incidence rate, understanding the co-morbidity between the two disease groups is necessary to properly assess, monitor, evaluate, and control their prevalence [1,2]. Cardiovascular diseases and their risk factors including diabetes mellitus (DM) are major contributors to the growing NCD burden [3]. WHO projects that DM will be the 7^th^ leading cause of death in 2030. Today there are 347 million people worldwide who have DM [4], around 90% of them type 2 DM [5]. More than 80% of DM deaths occur in LMIC[5]. In high income countries DM has long been known for its association with increased susceptibility to infections such as tuberculosis [6]. Although these associations have been attributed in part to DM associated alterations in innate immunity, related evidence is inconsistent and underlying mechanisms remain poorly understood. They are likely to vary by type of infection [7]. Yet, only few studies investigated the complex associations of diabetes with neglected tropical diseases (NTDs).

Dengue, one of 17 diseases assigned NTD status by WHO, is next to malaria the most important arthropo-borne (ARBO) tropical infection caused by the dengue virus. It is transmitted by several mosquito species within the genus *Aedes*, principally *A*. *aegypti* [8]. The number of Dengue virus infections has increased 30fold over the last decades. Today it is a major public health problem in tropical and subtropical regions [9]. The absence of adequate public health awareness, surveillance and control, population growth, globalization and urbanization contributed to this increase. An estimated 2.5 billion people are at risk of infection in over 100 endemic countries[10]. WHO estimates that currently between 50 and 100 million dengue infections occur annually. An estimated 500’000 dengue patients with potentially life threatening symptoms require hospitalization each year and about 2.5% of those affected die [11]. Dengue ranges from asymptomatic or self-limiting non-severe dengue (with or without warning symptoms) to severe dengue, characterized variously by severe plasma leakage, severe bleeding or severe organ involvement [12]. The studies reviewed in this paper were all except one conducted before 2009 and therefore routine clinical data was collected according to the WHO 1997 guidelines. These guidelines group symptomatic dengue virus infections into three clinical categories: undifferentiated fever, dengue fever (DF), and dengue hemorrhagic fever (DHF). DHF is further subclassified into four severity grades, with grades III and IV being defined as dengue shock syndrome (DSS) [13]. The clinical presentation of a dengue infection is difficult to predict, although the presence of warning signs occurring within 3 to 7 days after the first symptoms warrant strict observation and medical intervention. Intervention, including intravenous rehydration as the therapy of choice, can reduce case fatality in severe dengue to less than 1% [14]. Dengue has long been viewed as a pediatric disease, but the average age of dengue cases has been rising and there is a suggestion for adults to be at increased risk for dying from dengue. The increase of tourism in tropical regions also contributed to the increase in adult dengue cases [15].

There are five dengue virus (DENV) serotypes (DENV-1, DENV-2, DENV-3, DENV-4 and DENV-5), however, serotype 5 seems not to have a sustained transmission cycle in humans [16]. Infection confers immunity to the infecting serotype which is life-long, but not to the remaining three. Subsequent infections with a different dengue virus serotype increase the risk of severe complications [17]. Other than that, factors increasing the risk of severe clinical manifestations remain poorly characterized [18–20]. Present evidence suggests that beyond viral factors age, gender, social status, genetic background, sickle cell anemia, uremia, bronchial asthma, allergies, hypertension, chronic renal failure and also DM might adversely influence the clinical presentation of an infection [18,19,21–24].

In acknowledging the shift of dengue to older ages and the steep increase in the prevalence of DM, the objective of this review is to access the current clinical and epidemiological evidence for this NCD to contribute to a higher risk of a severe clinical presentation in dengue fever patients.

## Methods

### Search strategy

We conducted a systematic literature review using MEDLINE database to access any relevant publication describing an association between dengue and diabetes up to February 28 2014. The search terms used were “("dengue"[MeSH Terms] OR "dengue"[All Fields]) AND ("diabetes mellitus"[MeSH Terms] OR ("diabetes"[All Fields] AND "mellitus"[All Fields]) OR "diabetes mellitus"[All Fields] OR "diabetes"[All Fields]”.

### Inclusion criteria

We included articles in all languages; articles which reported on epidemiology, clinical signs, and laboratory parameters for dengue-infected patients, or on severity assessment. There was no restriction in publication dates, place of study, study design or age of research participants. As a validity assessment, the PRISMA criteria were used [25,26].

For meta-analyses, we included studies that compared the prevalence of DM between persons affected by different dengue stages (case-control studies), reporting estimates of association and their 95% confidence intervals, or enough information to derive this.

### Data extraction

We extracted the year during which dengue cases were diagnosed (year during which the study was conducted), year of the publication, country of the study, study design, study definitions of dengue infection and diabetes, and confounder adjustments. We extracted data on the sample size of enrolled persons and number of cases and controls and on the estimates (unadjusted and adjusted models) of the association (and their 95% confidence intervals) between diabetes and severe dengue. The transition of the patients data from clinical or hospital records to this review was based on published non-individual and non-identifying data. Data were extracted from the published papers independently by two reviewers and disagreements were resolved by discussion.

### Case definitions

#### Dengue

The studies included in this review mostly included dengue diagnosed before 2009 and applied the WHO 1997 dengue classification criteria [13]. A 2009 classification was proposed by the WHO/TDR group and in 2011 the WHO/SEARO group also suggested modifications [14]. We list the classification system applied in the respective studies in Table 1, which summarizes the available evidence. Control status and case status are predominantly defined as DF and as DHF or DSS respectively. Cases of DHF/DSS in the studies reviewed were hospitalized cases or deaths confirmed serologically, clinically with hemorrhagic manifestations or radiographically with a certain extent of plasma leakage. According to WHO 1997 classification, DHF is clinically subdivided into grades I-IV and all 4 grades could have some degree of at least subclinical plasma leakage. Grade I is the presence of fever with positive tourniquet test, grade II presents with spontaneous bleeding into the skin and elsewhere, grade III shows clinical signs of shock or circulatory failure, and grade IV presents with severe shock with undetectable blood pressure and pulse. Grade III and IV have also been labeled as “dengue shock syndrome” (DSS). The WHO 2009 classification differs from the 1997 classification scheme in that dengue cases are classified by the levels of severity with or without warning signs. Several clinical features specifically listed in the WHO 2009 classification were not directly obtained in the reviewed studies, e.g. clinical features such as lethargy, restlessness, and severe organ impairment or failure. We refer to DHF/DSS as “severe clinical presentation of dengue”.

**Table 1:** Summary of case-control studies and case series.

First author and publication date	Country & Year of study	Study design	Characteristics of Study population	Results(Odds ratios (OR) and 95% confidence intervals)
Epidemiological case-control studies				
Figueiredo et al.(2010)	Brazil, 2002–2003 (Salvador), 2003–2005 (Fortaleza)	Population-based case-control study	**Cases**:170 acute DHF	Predominantly DENV-3,Association DM—acute DHF vs. asymptomatic (IgG) (+)ve controls Adjusted OR (age, sex, income, neighborhood, skin colour, education)
			-registered in the national surveillance systems	DM yes vs. no.: aOR 2.75 (1.12–6.73)
			-residents of 2 cities	DM according to the number of medications: no medication vs. no DM: aOR 1.83 (0.18–18.67)
			-no age restriction	1 medicine vs. no DM: aOR 2.72 (0.86–8.60)
			-diagnostic criteria:	Insulin/ >1 medicine vs. no DM:aOR 3.36 (0.72–15.61)
			surveillance record review by 2 physicians;	DM prevalence: controls:2.6%,cases:5.3%
			Brazilian Health Service (very similar to WHO 1997) criteria;	
			fever & positive serology for anti-dengue IgM and/or viral isolation and characterization;	
			at least two signs or symptoms of dengue fever (headache or retroorbital pain, myalgia, arthralgia, prostration, exanthema);	
			all of the following signs: hemorrhagic manifestations, hemoconcentration with an increased haematocrit level; thrombocytopenia;	
			no consideration of ascites or pleural effusion (rarely recorded)	
			**Controls**: 1175	
			-neighborhood controls	
			-sero-positive (anti-dengue IgG)	
			-self-report of dengue like illness in same year as matched case	
			-no history of DHF	
			-matched for age and sex (within 5 years)	
			**Information on dengue history, confounders, effect modifiers & comorbidities**: in-person interviews with cases and controls	
			**DM**: self-report of physician diagnosis and verification of prescription /packaging of medication	
Mahmood et al (2013)	Pakistan (2011)	Hospital-based case-control study	**Cases**: 132 acute DHF	Association DM—acute DHF vs. asymptomatic IgG positive controls: Adjusted OR (sex, age, duration of illness)
			-admitted to two major tertiary care hospitals of Lahore	DM yes vs. no: aOR 1.26 (0.78–2.03), p = 0.34
			-age 15–65	DM according to its duration:
			-diagnostic criteria:	5–10 vs. <5 yrs aOR 2.76(0.77–9.84),p = 0.11
			diagnosed as DHF by a trained clinician;	>10 vs. <5 yrs:aOR 1.86 (0.55–6.26),p = 0.31
			WHO criteria (version not specified)	DM prevalence: controls: 42%, cases:43%
			**Controls**: 249 patients without acute dengue	
			-random sample of patients from same health facilities admitted for reasons other than dengue;	
			-positive for anti-dengue IgG;	
			-matched for age and sex (within 5 years);	
			-Information on dengue history, confounders, effect modifiers & co-moribities: in-person interviews with cases and controls; checklist for clinical record review	
			**DM**: self-report or clinical record	
Pang et al (2010)	Singapore (2006–2008)	Hospital-based case-control study	2006 epidemic: Cases:149 acute DHF, Controls:326 acute DF	2006 epidemic: predominantly DENV-1
			2007/2008 epidemic: Cases:590 acute DHF, Controls:1141 acute DF	27.6% of patients PCR (+)ve
			-admitted to the largest hospital of Singapore for dengue	72.4% of patients sero-positive & PCR(-)ve
			-adult	Association DM- acute DHF vs. acuteDF: Adjusted OR (age, ethnicity)
			-diagnostic criteria:	DM yes vs. no: aOR 0.34 (0.06–1.89)
			**probable DF**: positive acute dengue serology (Dengue Duo IgM & IgG Rapid Strip Test) and clinical criteria of DF by WHO1997;	DM prevalence: controls: 2.2%, cases: 1.3%
			**confirmed DF**: positive dengue PCR and clinical criteria of DF by WHO 1997	2007/8 epidemic: predominantly DENV-2
			-**DHF**: presence of all four criteria of fever, hemorrhagic manifestations, thrombocytopenia, plasma leakage	32.6% of patients PCR positive
			**Information on dengue history, confounders, effect modifiers & comoribities:** clinical record review	67.4% of patients sero-positive & PCR (-)ve
			**DM**: clinical records	Association DM- acute DHF vs. acute DF: aOR (age, ethnicity, gender, hypertension)
				DM yes vs. no: aOR 1.78 (1.06–2.97)
				DM with hypertension: aOR 2.16 (1.18–3.96)
				DM with hyperlipidemia:aOR 1.62 (0.90–2.92)
				DM with asthma: aOR 4.38 (0.80–23.85)
				DM prevalence: controls:3.5%, cases:6.4%
Lee et al. (2006)	Taiwan (2002)	Hospital-based case-control study	**Cases**:232 acute DHF (12 DSS)	Association DM- acute DHF/DSS vs. acute DF: Adjusted OR (factors not reported)
			**Controls**:412 acute DF	DM yes vs. no: aOR 1.86 (1.04–3.37)
			-all confirmed acute DF treated at Kaohshiung Medical University Hospital in 2002	DM prevalence: cases: 16.8%, controls:7.6%
			-no age restriction	Plasma leakage prevalence: Pleural effusion: 51% of DHF cases
			-diagnostic criteria for confirmed acute **DF**:	Ascites: 31% of DHF cases
			WHO 1997 criteria, meeting any of:	
			positive dengue virus by PCR;	
			4-fold increase of dengue virus-specific IgM or IgG in paired serum samples;	
			positive for dengue virus-specific IgM or IgG in a single serum sample;	
			additional diagnostic criteria for confirmed acute **DHF**:	
			Thrombocytopenia;	
			Evidence for hemorrhage and plasma leakage;	
			additional diagnostic criteria DSS:	
			hypotension, narrow pulse pressure, clinical signs of shock	
			**Information on dengue history, confounders, effect modifiers & comoribities:** clinical record review	
			**DM**: clinical records	
Karunakaran et al (2014)	India (2005–2008)	Hospital-based case-control study	**Cases**: 10 acute dengue patients, fatal	Association DM—mortality among confirmed acute dengue patients
			**Controls**: 40 acute dengue patients, non-fatal	DM yes vs. No: Crude OR 26.0 (2.5–273.7)
			-confirmed dengue patients admitted to in South Kerala hospital between 2005–2008	DM prevalence: controls:2.5%, cases:40%
			diagnostic criteria:	
			-confirmation by PCR or IgM antibody	
			**Information on dengue history, symptoms, confounders, effect modifiers & comorbities:** semi-structured interview; assessment of warning signs according to WHO 2009 case definition	
			**DM** mellitus: self-report	
**Case series**				
Lye et al. (2009)	Singapore (2004)	Hospital-based	**Cases**: 1971 acute DF, DHF, and DSS cases	DM prevalence: Age <60:2%, Age ≥ 60: 17%
			-all patients admitted to the Tan Tock Seng Hospital in Singapore in 2004	
			- fulfilling WHO 1997 criteria for acute dengue	
			- positive dengue diagnostic tests:	
			- probable dengue:(+)ve acute dengue serology (Dengue Duo IgM & IgG Rapid Strip Test)	
			- confirmed dengue:	
			- positive PCR	
			- no age restriction	
			**Information on dengue history, symptoms, confounders, effect modifiers & comoribities: clinical records**	
			**DM**: clinical records	
Wieten et al. (2012)	Netherlands (2006–2011)	Tropical and travel-medicine based case series	**Cases**: 132 acute dengue cases	DM prevalence: 8%
			-dengue patients serologically tested at Amsterdam Medical Center between 2006–2011	
			-diagnostic criteria:	
			serological confirmation:positive anti dengue IgM or at least fourfold increase in dengue specific IgG if possible based on one sample from the initial phase and one from the convalescent phase or else according to WHO 2009 criteria for a single sample;	
			clinical picture of probable dengue (WHO 1997 and 2009 criteria) or DHF (WHO 1997) or dengue with warning signs (WHO 2009);	
			**Information on dengue history, symptoms, confounders, effect modifiers & comoribities: clinical records**	
			**DM**: clinical records	
Sam et al (2013)	Malaysia (2006–2007)	Hospital-based	**Cases**: 10 acute DF/DHF/DSS patients, fatal	DM prevalence: 30% (pre-existing)
			-fatal cases at the University Malaya Medical Center 2006–2007	Plasma leakage prevalence: 78% of all cases
			-diagnostic criteria:	(67% among diabetic patients)
			laboratory confirmation: acute phase dengue-specific IgM and IgG; RT-PCR;	
			disease severity classification WHO 1997;	
			age range: 11–59;	
			**Information on dengue history, symptoms, confounders, effect modifiers & comoribities:** clinical records	
			**DM**: clinical records	
Lahiri et al (2008)	Singapore (2004–2005)	Hospital-based	**Cases**: 9 acute dengue cases, fatal	DM prevalence: 78%
			-fatal cases of a total of 1235 admissions with acute dengue in 2004–2005	Plasma leakage prevalence: 28% of DM patients
			-all 9 patients had positive laboratory test for dengue (7 by IgM, 3 by PCR)	
			-all 9 patients had evidence for capillary leakage and hemorrhagic manifestations	
			-7 of 9 patients strictly met DHF WHO 1997 criteria	
			- age range: 37–71	
			**Information on dengue history, symptoms, confounders, effect modifiers & comoribities**:	
			clinical records	
			**DM**: clinical records	
Hasanat et al.(2010)	Bangladesh (2009)	Hospital-based (prospective)	**Cases**: 133 acute dengue cases	Status on 1st OGTT (n = 133): Normal:25%, Glucose intolerant:54%, DM:21%
			-patients admitted to Samoritha Hospital in Dakha for dengue	Status on 2nd OGTT (n = 40):
			- laboratory confirmation by anti-dengue antibody test 1 week of onset of illness	Normal:83%
			-further diagnostic criteria not provided	Glucose intolerant:17%
			**Information on dengue history, symptoms, confounders, effect modifiers & comorbities:** interviews	Repeatedly normal:25%,
			**DM**: Oral glucose tolerance testing in consenting cases: 1st test between day 3 and 10 of admission and 2nd test before discharge	Repeatedly abnormal:18%
				Reverting to normal:55%

In the reviewed publications, laboratory diagnosis methods to confirm dengue virus infection as the cause of disease varied across studies and involved detection of virus, viral nucleic acid, antigens or antibodies, or a combination of these techniques [12]. The detection of virus, nucleic acid or antigen in the blood is confirmatory for an acute dengue infection. Antibody response to infection differs by the host immune status. In the setting of a primary infection, IgM are the first antibodies to appear, but it takes 10 days for them to be detectable in 99% of patients. During a secondary dengue infection, anti-dengue IgG are detectable at high levels already in the acute phase, whereas IgM levels remain significantly lower than in primary dengue infection. A four-fold or greater increase in IgG antibody levels in paired sera (sample collected during the acute stage of illness and sample collected in the convalescent stage) indicates acute dengue [12]. Identification of virus/viral RNA/viral antigen and antibody response are ideally combined for the confirmation of acute dengue. Yet, as specimen collection and processing is easier for serology, the laboratory methods applied in the studies mostly conducted in LMIC varied broadly. We list the laboratory confirmation methods applied in the respective studies in Table 1, together with the available evidence.

#### Diabetes mellitus

In studies included in this review, the diagnosis of DM was mostly ascertained by self-reported diagnosis in the context of in-person interviews or DM diagnosis listed in clinical records. The DM diagnosis was further verified by requesting the prescription and/or medication package during the in-person interview in the only population-based case-control study [27]. An oral glucose tolerance test was performed in dengue patients in the case series published by Hasanat study [28].

### Meta-analysis

We used random-effects models for meta-analyses of the association between DM and a severe clinical presentation of dengue [29]. They take into consideration the variation between the true effects estimated by included studies unlike fixed effect models which assume a common true effect across studies. We used odds ratios as measure of association across all studies. We used the estimates reported by the authors as “primary model” and the I^2^ metric and Tau^2^ to describe the between study heterogeneity and variance respectively.

We conducted sensitivity analyses by using a fixed effect model and excluding a study, which was based on non-matched random controls and that only reported unadjusted estimates [30]. We performed analyses with Stata version 12 (Stata Corporation, Texas) and considered p<0.05 as statistically significant.

## Results

**Fig 1:**
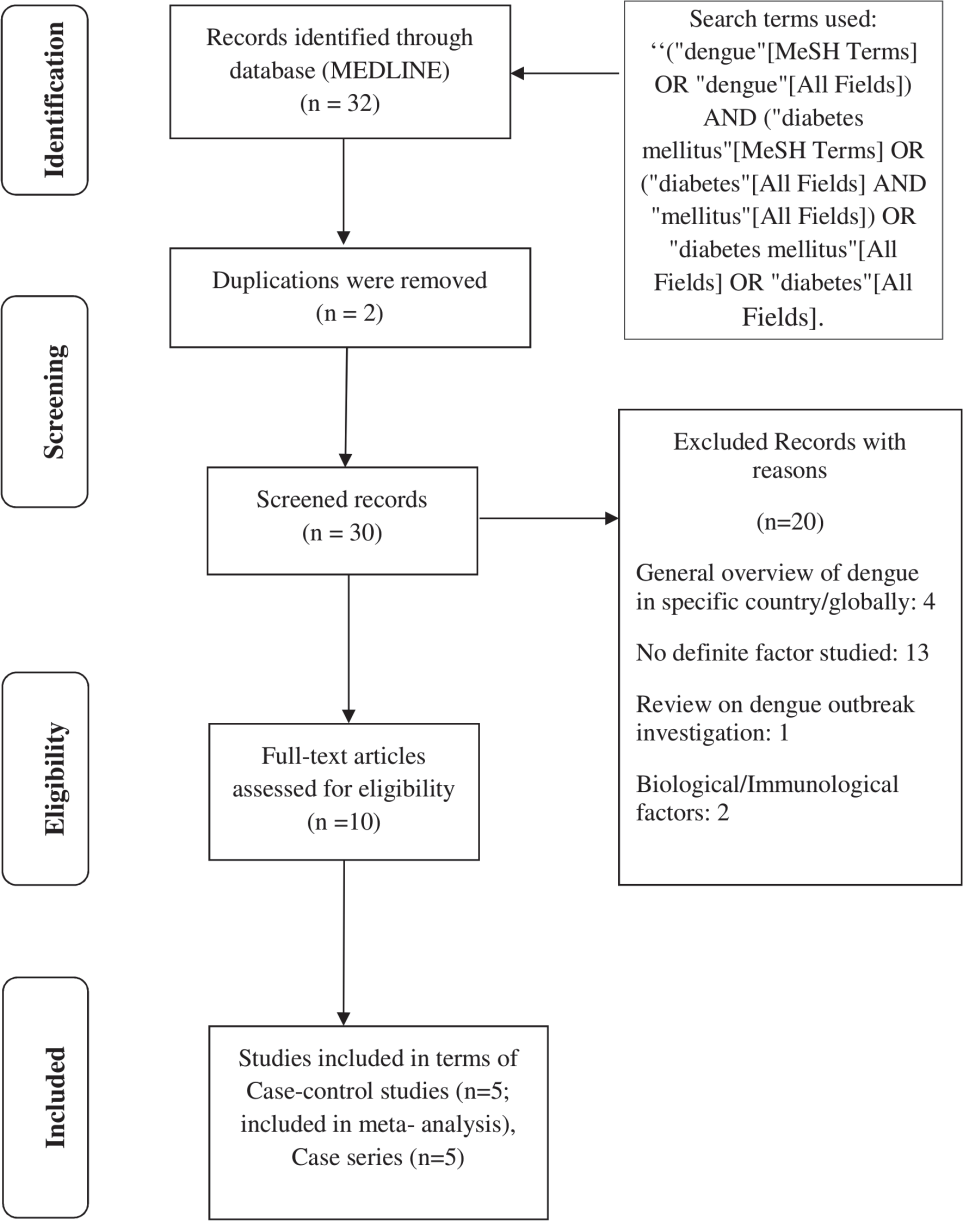
PRISMA flow diagram of diabetes and dengue.

Our literature search resulted in 32 hits (Fig 1). After excluding duplicates and non-relevant articles based on a full text analysis (single case reports, outbreak investigation reports, no specific factors studied, narrative overview of dengue infections in a specific country or globally), ten articles were retained. Among them, five studies were case-control studies [30–33] with one study being population-based [27]. The case-control studies compared the prevalence of DM in persons with acute or past non severe dengue (controls) to that in persons with an acute severe clinical presentation of dengue. Five additional articles (case series) characterized DM in mostly dengue patients with severe clinical manifestations, including fatal cases [28,34–37]. These studies lack a comparison group with acute or past non severe of dengue, and are therefore not included in the meta-analysis.

### Epidemiological evidence from case-control studies on the association between DM and a severe clinical presentation of dengue

Epidemiological studies included in the meta-analysis compared the prevalence of DM and other co-morbidities between patients suffering from acute dengue with a severe presentation and controls with acute or past dengue without severe clinical manifestations (Table 1). In the absence of controls without evidence for a dengue infection history, studies thereby compared the prevalence of DM in dengue patients with different degrees for severity in clinical presentation, rather than the risk of being infected with dengue virus.

The only population-based case-control study [27] identified was conducted in two Brazilian cities and included both, children and adults. DHF cases were ascertained through the national surveillance system. The surveillance records were reviewed by two physicians. DHF was defined according to criteria used by Brazilian Healthy System, which were very close to WHO 1997 criteria. Controls were selected from the same neighborhood as cases. In addition, they were tested positive for anti-dengue IgG and matched to cases by age, sex and a report of a past dengue-like fever in the same year as the DHF diagnosis of cases. Additional information from both, cases and controls was obtained through in-person interviews. DM was based on a self-reported physician-diagnosis. Interviewers also asked for medication intake and verified it by seeing the prescription of packaging. DM was statistically significantly associated with DHF independent of age, sex skin color, income and educational level (aOR 2.75, 95% CI 1.12–6.73). The association of self-reported diabetes with DHF was stronger in diabetic patients being treated, especially if treated with insulin or more than 1 drug (aOR 3.36; 95% CI 0.72–15.61). In addition, white ethnicity (aOR 4.70, 95% CI 2.17–10.2), high income (aOR 6.84, 95% CI 4.09–11.43), high educational level (aOR 4.67, 95% CI 4.09–11.43) and a self-report of allergy treated with steroids (aOR 2.94, 95% CI 1.01–8.54) were also associated with a more severe clinical presentation of dengue.

A hospital-based study including persons aged 15 to 65 in Pakistan [31] compared hospitalized acute DHF patients (cases) to dengue IgG positive patients, but hospitalized for unrelated conditions (controls). The study was conducted in two major tertiary care hospitals. A DHF case was defined as diagnosis by an experienced clinician applying WHO criteria, although the version applied was not specified. The categorization into persons with and without DHF points to the use of WHO 1997 criteria. Information was obtained through structured record review and in-person interview. Age- and sex-matched DHF cases were slightly more likely to report a DM diagnosis than control patients. In both groups, the reported prevalence of DM was exceptionally high, 41.8% in controls and 43.2% in cases. The association of DM with DHF adjusted for age, sex and duration of illness was statistically non-significant (aOR 1.26, 95% CI 0.78–2.03, p = 0.34).

In Singapore, a hospital-based study was conducted in the nation’s largest clinic. It included all admitted patients with acute dengue without age restriction [32]. Information on case and control status as well as comorbidities was exclusively derived from abstracting medical charts. Cases were defined as DHF patients, and controls were DF patients. Probable dengue patients had a positive acute dengue serology. Confirmed dengue patients had positive dengue polymerase chain reaction assays. Clinical diagnosis for DHF was based on WHO 1997 criteria. The data was analyzed separately for the two epidemic periods of 2006 (predominantly DENV-1) and 2007/2008 (predominantly DENV-2; larger sample size). No association between DM status and DHF was found in the 2006 dengue outbreak. DM was independently associated with DHF in the 2007/8 epidemic (aOR 1.78, 95% CI 1.06–2.97). The association was stronger if the diabetic patients additionally had hypertension (aOR 2.16, 95% CI 1.18–3.96) or asthma (aOR 4.38, 95% CI 0.80–23.85). Mean hospitalization days were longer for DM (4.99±3.34days) as compared to non-DM patients (4.04±1.62 days, p = 0.001). Additional factors associated with DHF were Chinese ethnicity (compared to Malay or Indian ethnicity) (aOR 1.90, 95% CI 1.01–3.56 in 2006 epidemic periods and aOR 1.67, 95% CI 1.24–2.24 in 2007/2008 epidemic periods) as well as middle age in 2007/2008 (aOR 1.41, 95% CI 1.09–1.81 in 30–39 years and aOR 1.34, 95%CI 1.09–1.81 in 40–49 years of age group).

In 2002 in Taiwan, all patients with confirmed acute DF treated at the Kaoshiung Medical University Hospital during a large outbreak occurring in the southern Taiwan were categorized into groups of DF and DHF/DSS by strictly adhering to clinical WHO 1997 criteria and laboratory confirmation under the auspices of the Taiwanese Centre of Disease Control [33]. Clinical information such as signs and symptoms and the results of blood investigation were abstracted from medical records. Cases were mostly adults, only 4.5% were below age 15 years. The prevalence of DM was 16.8% in DHF/DSS cases compared to 7.6% in DF patients (controls). The adjusted OR reported was 1.86 (95%CI 1.04–3.37), albeit covariates were not reported. In addition to the independent association of DHF/DSS with DM, statistically significant associations with hypertension and renal insufficiency, uremia, past history of dengue infection as well as male gender and older age were also found.

A hospital-based study in Southern India [30] obtained information on patients admitted to the largest multi-specialty hospital in South Kerala for acute dengue between 2005 and 2008. The case group consisted of 10 in-hospital deaths of patients admitted with a clinical diagnosis of probable dengue, which was confirmed by either RT-PCR or IgM antibody tests, and review of clinical symptoms through medical record review. Forty non-matched controls were randomly selected among patients with a confirmed acute dengue, but recovering from the illness. The classification of dengue among the controls was not specified. Information on co-morbidities and other factors was abstracted from medical records. The prevalence of DM in controls was 2.5% compared to 40% in cases. DM was a strong predictor of mortality in the bivariate analysis (OR 26.0, 95% CI 2.47–273.67, p = 0.004). In the same study, hypertension was also a strong predictor of mortality (OR 44.3, 95% CI 6.2–315.5, p = 0.000). Mortality was much higher in patients over 40 years (OR 9.3, 95% CI 1.9–44.4, p = 0.002). No adjusted odds ratios, which would facilitate the interpretation of independency in the reported associations, were reported.

### Meta-analysis of epidemiological evidence from case-control studies

We included the results of five above mentioned studies in a meta-analysis. One of these studies reported two separate estimates from two independent cross-sectional assessments; hence, we considered them as separate studies. The meta-analysis showed that the presence of a severe clinical presentation of dengue was positively associated with the presence of DM.

**Fig 2:**
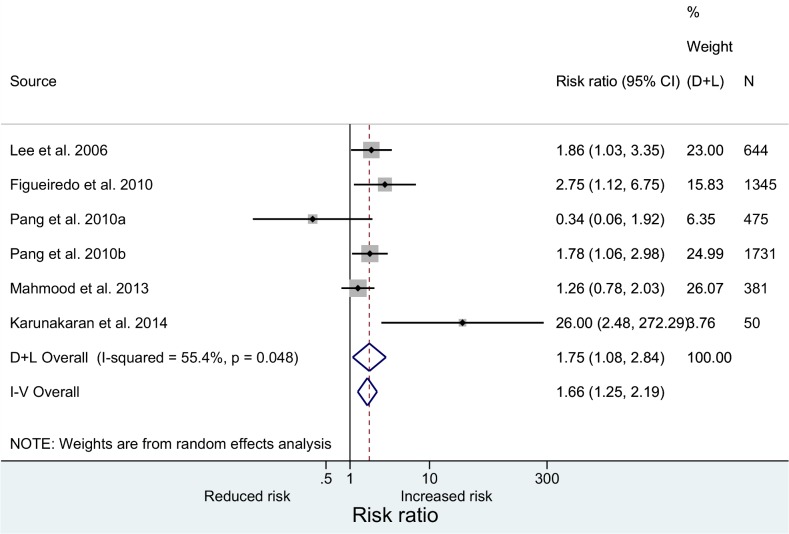
Meta-analysis of case-control studies on the association between diabetes mellitus and a severe clinical presentation of dengue.

A diagnosis of DM was associated with an increased risk for severe clinical manifestations of dengue by 75% (95% CI: 1.08–2.84, p = 0.022) compared to non-DM patients (Fig 2). This OR remained robust across sensitivity analyses involving fixed effect analysis. We observed some heterogeneity across the studies, consistent with the broadly differing study settings. The small number of studies included in the meta-analysis did not provide statistical power for formal statistical assessment of heterogeneity (Fig 2).

### Clinical case series on characteristics of DHF patients

We additionally identified five case series reporting dengue-related hospitalizations and the prevalence of DM in these cases. They are listed in Table 1 as reference of the prevalence of DM in dengue patients with a severe clinical presentation. The studies have been conducted mostly in Asian dengue endemic regions such as Malaysia [36]; Bangladesh [28]; Singapore [34,35]. In the absence of a control group, these studies are of limited value for better understanding of the role of DM on the clinical presentation of dengue. In several instances the case definition was restricted to just being dengue seropositive. This does not allow differentiating between DM influencing the clinical manifestations of dengue infection versus dengue infection influencing the clinical presentation of DM. Of interest, in that respect is the study by Hasanat et al [28]. Hospitalized DF patients underwent oral glucose tolerance testing (OGTT) between 3 and 10 days after the start of illness. A subset of these patients agreed to a second OGTT before discharge. The authors demonstrated a high rate of glucose intolerance in the early phase of disease, which returned though to normal in 55% of the patients.

## Discussion

The few published studies specifically addressing the role of DM as a risk factor for a severe clinical presentation of dengue provide suggestive evidence for an adverse effect. This result merits further investigation in the context of study designs that overcome the weaknesses of currently available publications as addressed below.

Assigning causality to the modifying effect of diabetes on the clinical presentation of dengue is premature. First, the case-control studies conducted to date and summarized above are mostly retrospective in nature. The clinical and laboratory diagnostic criteria applied in different studies vary broadly, as does the definition for the control group. Second, the WHO 1997 dengue classification used in the studies reviewed, has itself a number of limitations. For example, the development of this classification was based on disease patterns of children in Thailand, potentially limiting its generalizability to other geographical regions and to older age groups. Furthermore, clinical assessments such as the Tourniquet test do not differentiate between DF, DHF and other febrile illness [38,39]. It also fails to detect severe dengue manifestations in many patients [40]. Third, information on DM was either self-reported or record-based and did not systematically allow differentiating between DM diagnosed before versus concurrent with the dengue episode. Given the high degree of under diagnosis of DM, especially in LMIC, misclassification of DM status in the studies is likely substantial. The problem of under diagnosis is not easy to overcome, as DM measured at the time of acute dengue is not necessarily reflecting the underlying DM status, but rather stress-related hyperglycemia, which disappears after recovery from dengue [28]. Longitudinal studies of dengue patients are needed that allow studying both, the longitudinal course of hyperglycemia and the evolvement of clinical presentation of the dengue infection. In addition, whether better control of DM in early stages of dengue will prevent severe forms of dengue needs to be assessed in the context of intervention studies. The basis should be dengue surveillance programs which registers all dengue episodes and which includes routine collection of blood specimens for HbA1c and other hyperglycemia parameters and of information regarding diagnosis and treatment of DM. A fifth limitation of studies conducted to date is the fact that most of them were hospital-based case-control studies with a high potential for selection bias. But the results from the only population-based case-control study also point to DM increasing the risk for a severe clinical presentation of dengue [27]. Finally, study limitations also include the fact that the modifying effect of DM on different dengue serotypes could not be differentiated; that the age range of study subjects was not always reported; and that many studies included children and adolescents in whom DM is rare.The observed heterogeneity between the results of the individual studies is a limitation in the meta-analysis and reflects differences in the diagnostic and classification criteria for dengue and DM, differences in the controls selected, as well as differences in study design such as sample size, population and study setting or confounders considered in the analysis. Our systematic and broad search strategy, (not limiting to publication dates, place of study, study design or age of research participants) and the novelty of this topic are the strengths of our review and should stimulate further research into the topic, also in the light of supporting results from experimental studies.

Biological evidence exists to support the hypothesis for a high DHF: DF ratio in diabetes. One example for a line of reasoning is that the cytokine overload related to a Th1 to Th2 shift and a severe manifestation of dengue superimposed by the cytokine overload of a diabetic state may be particularly detrimental to the endothelium and for subsequent vascular leakage [41–43]. The third space fluid shift such as pleural/pericardial effusion or ascites is an important clinical manifestation in dengue with severe clinical symptoms, which is a consequence of endothelial dysfunction and results in hemoconcentration, hypotension and shock [34]. DM shares with a severe clinical presentation of dengue alterations in the innate immune response, a pro-inflammatory state and endothelial dysfunction. Lee and colleagues infected mononuclear cells from diabetic (n = 33) and healthy individuals (n = 29) with dengue virus. Cells from diabetics produced higher levels of IL-4 and of both, IL-4 and IL-10 as well as granulocyte-macrophage colony-stimulating factor (GM-CSF) on the first and third day post-infection, respectively. No differences in viral load were found [44]. In a post-mortem study of DHF/DSS related deaths, the intestinal serosa of 1 DM case with ascites, but not of non-DM cases showed apoptosis of microvascular endothelial cells [42]. Microvascular endothelial cells cultured with sera from acute dengue infection patients containing high TNF-α levels exhibited activation and apoptosis, a pathophysiological alteration likely related to vascular leakage [41].

Currently available epidemiological studies on the DM/DHF, DSS association do not provide sufficient clinical details to specifically address this “cytokine overload” hypothesis. For example, the Brazilian study explicitly stated that it did not look into ascites or pleural effusion, as these were very rarely recorded [27]. The other studies did not mention plasma leakage such as pleural effusion or ascites except for the Taiwan study, where 51% of 164 DHF cases showed evidence of pleural effusion in X- ray and 31% of DHF cases had ascites in ultrasound [33]. Future studies thus need to specifically address the issue of plasma leakage in the overlap between severe dengue and DM. Clinical reporting of the presence of pleural effusion and ascites as signs of severe plasma leakage is essential, as hemoconcentration and shock symptoms in the absence of plasma leakage can also arise from poorly controlled DM. The direct hypovolemic effect of hyperglycemia and the associated elevated hematocrit may even lead to misclassification of diabetics as DHF in the absence of objective evidence of plasma leakage. While recognition of hemoconcentration irrespective of its reason is of primary clinical importance for the provision of appropriate fluid therapy, currently available studies on the DM and DHF/DSS association do not provide sufficient clinical details to differentiate between effects of DM vs. DHF/DSS.

The potential role of altered innate immunity in mediating the association between DM and a severe clinical presentation of dengue obtains indirect support by observations that the latter may also have a higher prevalence of asthma and allergies. Patients with asthma and allergy also exhibit altered Th1 and Th2 responses [27,31]. But in DM, factors beyond the altered cytokine profile may additionally put people at risk. Diabetic patients express high rates of hypertension and impaired renal function which are both themselves associated with endothelial dysfunction [45,46]. Hypertension was also observed to be more common in patients with dengue [27,30–32,35], but often exhibited no independent effect in regression models adjusting for diabetes [31,32]. Pang and colleagues (2012) reported a higher risk for a severe clinical presentation of dengue in hypertensive when compared to non-hypertensive diabetic patients [32].

### Conclusion

Understanding factors increasing the likelihood of dengue patients with severe clinical symptoms would help the physician to decide in a timely fashion on the need for close observation, adequate treatment, or hospitalization. The available evidence points to DM as a potentially important co-factor. Additional prospective studies among DM and non-DM patients are needed to assess the impact of pre-existing DM as well as of hyperglycemia at the time of dengue diagnosis on the risk of a severe clinical presentation of dengue and of related deaths. Even in the absence of causal inference, it seems justified that fever episodes in patients with DM and living in a dengue endemic region are confirmed for dengue as soon as possible and that they remain under close surveillance if an acute dengue infection is confirmed. Future studies need to also address whether better control of glycemia level in dengue patients with DM can improve the outcome of the patient and decrease the risk of a severe clinical presentation. As the fluid management in diabetic patients with a severe clinical presentation of dengue poses a particular challenge, this issue should be taken into consideration by these studies. Diabetic patients in dengue endemic regions should consistently receive recommendations to protect against dengue infection by taking preventive measures against indoor mosquito breeding and against mosquito bites.

## Supporting Information

S1 Table PRISMA checklist. (PDF)
