# Supplementary material for: Is Diabetes a Risk Factor for a Severe Clinical Presentation of Dengue? - Review and Meta-analysis
Source: PLoS Negl Trop Dis. 2015 Apr 24;9(4):e0003741. doi: 10.1371/journal.pntd.0003741 (PMC4409149; doi:10.1371/journal.pntd.0003741)
Supplement: S1 Table — (PDF) [file pntd.0003741.s001.pdf]

Table S1. PRISMA Checklist

Section/topic	#	Checklist item	Reported on page #
**TITLE**			
Title	1	Is Diabetes a risk factor for a severe course of Dengue Fever Virus infection?-Review and Meta-analysis	1
**ABSTRACT**			
Structured summary	2	Abstract structured into:<br>-Background<br>-Methodology/Findings<br>- Conclusions/Significance	2
**INTRODUCTION**			
Rationale	3	Introduction section, Paragraphs 1-4	4-5
Objectives	4	Introduction section, paragraph 5	5
**METHODS**			
Protocol and registration	5	N/A	N/A
Eligibility criteria	6	Articles:<br>- with all languages<br>- articles reporting on epidemiology, clinical signs, and laboratory parameters for dengue-infected patients<br>- no restriction in publication dates, place of study, study design or settings and age of research participants. (Paragraph 2/Inclusion criteria subsection)	6
Information sources	7	MEDLINE with last date searched in Feb 28 2014. (Paragraph 2)	6
Search	8	The search terms used were ‘‘("dengue"[MeSH Terms] OR "dengue"[All Fields]) AND ("diabetes mellitus"[MeSH Terms] OR ("diabetes"[All Fields] AND "mellitus"[All Fields]) OR	6

		"diabetes mellitus"[All Fields] OR "diabetes"[All Fields]. (Paragraph 1/Search Strategy subsection)	
Study selection	9	The eligible studies were selected by following PRISMA guideline. For meta-analyses, we included studies that compared the prevalence of diabetes between persons affected by different dengue stages (case-control studies), reporting estimates of association and their 95% confidence intervals, or enough information to derive this. (Paragraph 4)	6
Data collection process	10	We extracted study year of publication, place and year of study, study setting and design, study definitions of diabetes and dengue infection, and confounder adjustments, data on the estimates (unadjusted and adjusted models) of the association (and their 95% confidence intervals) between diabetes and DHF. Data was independently extracted by two reviewers and disagreements were solved by discussion. (Paragraph 5)	6
Data items	11	Name of authors, year of publications, country and place of study, year of study, nature of study, characteristics of study populations (numbers, age of study participants),measures of outcomes and results(OR, 95% CI and p values) (Paragraph 5 of Page 6, Paragraph 1 of Page 7 and Table 1)	6,7, 23-28
Risk of bias in individual studies	12	Documentation of factors with a potential to bias results from individual studies in:<br>- laboratory confirmation for acute dengue<br>- WHO criteria for severity stage applied<br>- source for classification of diabetes state<br>- selection of cases and controls<br>- covariates considered in case-control matching and statistical analysis of the association between diabetes and severe dengue<br>(Paragraph 2-3 of page 7, 2-4 paragraph of page 8)	7-8
Summary measures	13	Odd ratio (OR) (Paragraph 3)	8
Synthesis of results	14	Random effect meta-analysis of case-control studies<br>(Paragraph 3-4)	8

Section/topic	#	Checklist item	Reported on page #
Risk of bias across studies	15	1) Documentation of divergent factors with a potential to bias results across studies in:<br>- laboratory confirmation for acute dengue<br>- WHO criteria for severity stage applied<br>- source for classification of diabetes state<br>- selection of cases and controls<br>- covariates considered in case-control matching and statistical analysis of the association between diabetes and severe dengue<br>2) Sensitivity analysis in meta-analysis: Sensitivity analyses:<br>- fixed effect meta-analysis of case-control studies<br>- meta-analysis omitting unadjusted study results<br><br>(Paragraph 2-3 of page 7, 2-4 paragraph of page 8)	7-8
Additional analyses	16	N/A	
**RESULTS**			
Study selection	17	Paragraph 1	9
Study characteristics	18	Name of authors, year of publications, country and place of study, year of study, nature of study, characteristics of study populations (numbers, age of study participants),co-morbid conditions, definitions of cases and controls, measures of outcomes and results (RESULTS section &Table 1)	9, 23-28
Risk of bias within studies	19	Detailed documentation of factors with a potential to bias study results in Text under the subsection of epidemiological case control studies and clinical case series and in Table 1<br><br>RESULTS section & Table 1	9-11,23-28

Results of individual studies	20	The results from case-control studies and case series are presented in the result section, paragraphs 1-6 as well as in a structured manner in Table 1<br><br>The results from the meta-analysis of the case-control studies are presented in Figure 2. The results from the sensitivity analyses are presented in the meta analysis of case control studies subsection in result section.	12,23-28
Synthesis of results	21	A diagnosis of type 2 DM increases the risk of DHF by 75% (95%CI: 1.08-2.84, p=0.022) compared to non-type 2 DM. (Paragraph 4)	12
Risk of bias across studies	22	Detailed documentation of factors with a potential to bias study results in Text under the subsection of epidemiological case control studies and clinical case series and in Table 1<br><br>(RESULTS section & Table 1)	9-11,23-28
Additional analysis	23	N/A	
**DISCUSSION**			
Summary of evidence	24	Discussion section, paragraph 1	13
Limitations	25	Discussion section, paragraphs 3 of page 14, Paragraph 2 of page 15	14-15
Conclusions	26	Separate conclusion section<br><br>.	16
**FUNDING**			

Funding	27	N/A	
